# Disseminated Cryptococcus gattii infection preceding onset of pulmonary alveolar proteinosis

**DOI:** 10.1002/rcr2.357

**Published:** 2018-08-02

**Authors:** Jessica Quah, Teck Boon Low, Raymond Fong

**Affiliations:** ^1^ Department of Respiratory and Critical Care Medicine Changi General Hospital Singapore; ^2^ Department of Infectious Diseases Changi General Hospital Singapore

**Keywords:** Anti‐GM CSF antibodies, crazy‐paving, cryptococcoma, pulmonary fungal infections, whole lung lavage

## Abstract

A 50‐year‐old immunocompetent man presented with intracranial space‐occupying lesions and a right lung mass. This was found to be disseminated Cryptococcus gattii infection. Following 15 months of anti‐fungal therapy, imaging showed reduction in the size of the pulmonary cryptococcoma and new multi‐lobar ground‐glass opacities interspersed with a crazy‐paving pattern. Surgical lung biopsy was performed after bronchoscopic evaluation was non‐yielding. Histology showed intra‐alveolar accumulation of foamy macrophages and airspaces containing periodic acid Schiff‐positive amorphous eosinophilic material with strong immune positivity for surfactant A, consistent with a diagnosis of pulmonary alveolar proteinosis (PAP). The majority of adult‐onset PAP is due to the presence of anti‐granulocyte macrophage colony‐stimulating factor antibodies. Opportunistic fungal and mycobacterial infections are known to occur in these patients due to alveolar macrophage and neutrophilic dysfunction. The onset of PAP may occur concurrently with, or be temporally distinct from, opportunistic infections. For patients with respiratory failure, whole lung lavage is a therapeutic strategy.

## Introduction

*Cryptococcus gattii* are encapsulated yeasts that can cause disseminated cryptococcosis in immunocompetent adults. Sites of diseases are predominantly pulmonary and cerebral. These fungal organisms can be found in tropical regions, such as Southeast Asia, and are associated with exposure to environmental sources such as gum trees, fir, and cedar. It may also be found in the air, soil, and water of heavily infested areas 1. It is less common compared to *Cryptococcus neoformans*, which is frequently found to cause disseminated disease in immunocompromised hosts.

Opportunistic infections are known to occur in pulmonary alveolar proteinosis (PAP). Here, we report a patient who had disseminated *C. gattii* infection before the onset of PAP.

## Case Report

**Figure 1:**
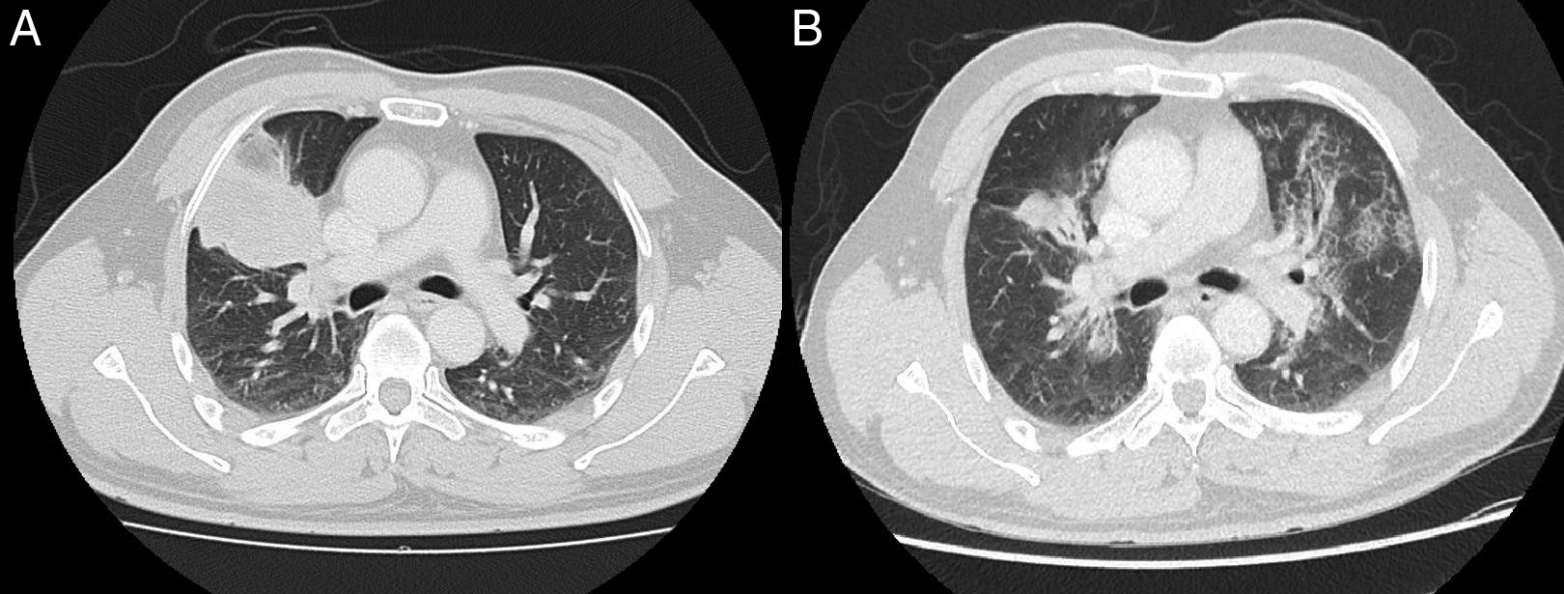
(A) Computed tomography (CT) thorax with right upper lobe pulmonary cryptococcoma at initial presentation. (B) CT thorax 15 months after initial of anti‐fungal therapy, showing decreased size of right upper lobe pulmonary cryptococcoma and development of new crazy‐paving pattern.

A 50‐year‐old Chinese man with no prior illnesses presented with a history of one month of fever, headache, and vomiting. Magnetic resonance imaging (MRI) of the brain showed multiple varying sizes of ring‐enhancing lesions scattered in both cerebral and cerebellar hemispheres. An initial diagnosis of metastatic brain tumour or infection was made. Computer tomographic (CT) scan of the thorax revealed a 6.5 cm × 4.5 cm right upper lobe mass that extended to the right hilum, radiographically suspicious for primary lung malignancy (Fig. 1A).

There was mild leucocytosis of 11.7 × 10^3^/uL and a normal C‐reactive protein of 1.5 mg/L. Lumbar puncture demonstrated raised intracranial pressure of 26 cm H_2_O with drainage of clear cerebrospinal fluid (CSF). There was an elevated cell count of 260 cells/mm^3^ of fluid with 90% lymphocyte predominance, low glucose of 1.9 mmol/L, and raised protein at 1.15 g/L. Initial mucicarmine and India Ink staining did not demonstrate organisms on staining. Subsequently, CSF cryptococcal antigen was detected by qualitative testing, and CSF fungal culture grew *C. gattii.* Blood cryptococcal antigen was detected at a titre of 1:1280. Of note, human immunodeficiency virus testing was negative.

**Figure 2:**
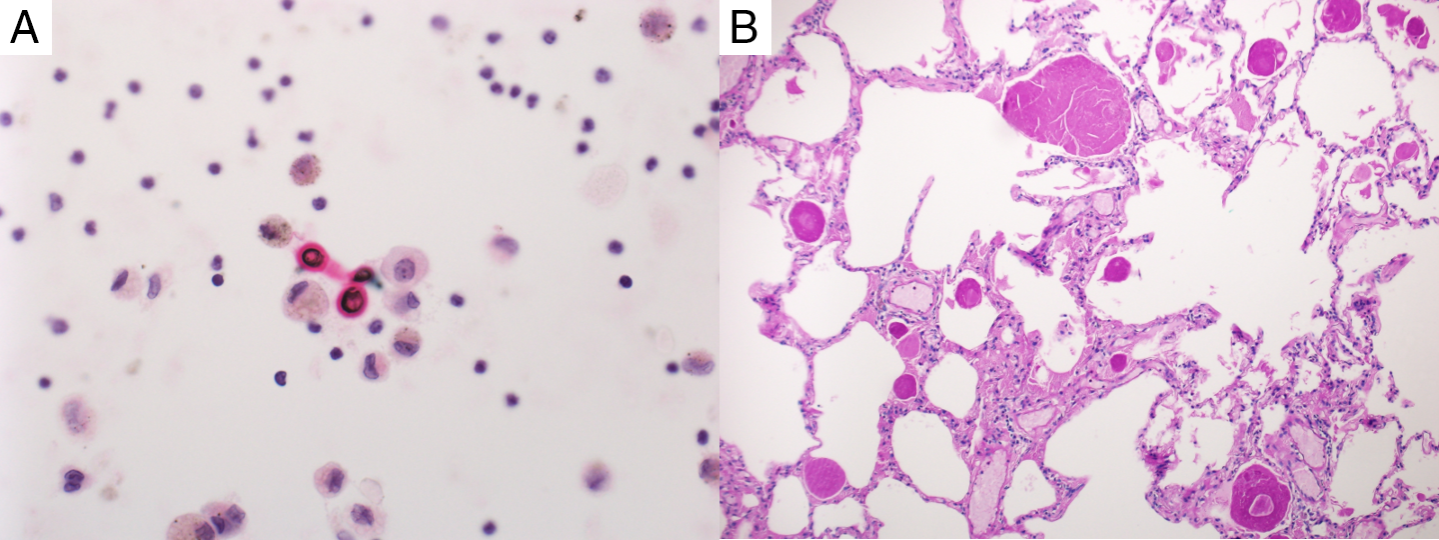
(A) Mucicarmine staining for yeasts showing Cryptococcus gattii in bronchoalveolar lavage. (B) H&E stain of lung biopsy demonstrating pulmonary alveolar proteinosis with intra‐alveolar accumulation of periodic acid Schiff‐positive surfactant.

Bronchoscopy was performed for evaluation of the right upper lobe mass. Bronchoalveolar lavage showed thick‐walled fungal yeast forms with narrow‐based budding, morphologically consistent with Cryptococcus yeasts. These were highlighted by mucicarmine special stain (Fig. 2A). Bronchoscopic biopsies showed non‐specific chronic inflammatory infiltrates in the submucosal stroma of the bronchial wall epithelium and lung parenchyma.

The patient received induction anti‐fungal therapy with four weeks of amphotericin B and two weeks of flucytosine. This was followed by maintenance therapy with oral fluconazole. Therapeutic lumbar punctures were performed for the treatment of raised intracranial pressure. On outpatient review, brain imaging showed cerebral cryptococcomas completely resolving at five months of therapy. CT chest performed for monitoring of right upper lobe cryptococcoma demonstrated reduction in the size of consolidation. However, at 15 months of anti‐fungal therapy, new ground‐glass opacities were seen in all lobes, with focal areas of crazy‐paving pattern seen in bilateral upper lobes (Fig. 1B). This occurred in tandem with the complaint of a new cough. Differential diagnoses include drug pneumonitis, interstitial pneumonias, and new opportunistic pulmonary infections.

Flexible bronchoscopy was repeated for evaluation of new opportunistic infections, but there were none detected. Bronchoalveolar lavage returned turbid fluid, occasional alveolar macrophages were seen, and periodic acid Schiff (PAS) stains did not reveal any abnormalities or fungal elements. Transbronchial lung biopsies yielded normal lung parenchyma.

A decision was made to use video‐assisted thoracoscopic lung wedge biopsy. Histological examination of the lung parenchyma showed intra‐alveolar accumulation of foamy macrophages and airspaces containing PAS‐positive amorphous eosinophilic material with strong immune positivity for surfactant A (Fig. 2B). This was consistent with a diagnosis of PAP. At the time of this report, the patient has preserved spirometric values, lung volumes, and corrected diffusion capacity of the lung for carbon monoxide (DLCO) of 90%. Plans have been made for monitoring of pulmonary function, with a view to commence therapeutic whole‐lung lavage when required.

## Discussion

Pulmonary alveolar proteinosis may be congenital, secondary, or acquired. Congenital PAP presents in neonates due to disorders of pulmonary surfactant production. In adults, secondary PAP occurs in individuals with a high level of mineral dust exposure, resulting in alveolar macrophage dysfunction; 90% of adult PAP is due to the presence of antibodies to granulocyte macrophage colony‐stimulating factor (GM‐CSF), which is normally required for alveolar macrophage terminal differentiation. The presence of anti‐GM‐CSF antibodies causes macrophage dysfunction, impairing the clearance of pulmonary surfactant in the alveoli and opportunistic infections 2. Inoue *et al*. described a large cohort of Japanese patients diagnosed with PAP, where 5.7% had concurrent pulmonary infections with aspergillosis, atypical mycobacteria, and mycobacteria tuberculosis. Opportunistic infections may precede the onset of PAP 3. Punatar *et al*. reviewed 75 case reports of opportunistic infections occurring in patients with PAP and found that 40% had infection preceding the onset of PAP by a mean of 17 months 4. For the patient in our report, anti‐GM‐CSF antibodies were not performed due to unavailability of the test.

Management strategies for PAP include therapeutic whole‐lung lavage for patients with respiratory failure, experimental recombinant GM‐CSF replacement, and lung transplantation. Zhao *et al*. performed a retrospective study on the eight‐year outcomes of Chinese patients with autoimmune PAP 5. Whole‐lung lavage was performed if any of following criteria were met: resting PaO_2_ < 65 mmHg, A‐a gradient >40 mmHg, or shunt fraction>10%; 32.5% of patients achieved remission or remained stable without requiring whole‐lung lavage, while 20% of patients required more than one lavage. DLCO of less than 42% may predict if patients required more than one lavage.

In summary, PAP and opportunistic infections have a clear association due to a common underlying disease mechanism. Increased clinical awareness may help to identify these associated entities as they may be temporally distinct.

### Disclosure Statement

Appropriate written informed consent was obtained for the publication of this case report and accompanying images.
